# Survey of Vaccine Hesitancy in Patients Visiting Three Tertiary-care Emergency Departments in Southeast Louisiana

**DOI:** 10.5811/westjem.57449

**Published:** 2023-09-14

**Authors:** Denrick Cooper, David Harmon, Carmel Alemayehu, Julia Levy, Mariella Gastañaduy, Lisa Birdsall Fort, Nicole McCoin

**Affiliations:** * Ochsner Health, Department of Emergency Medicine, New Orleans, Louisiana; † Ochsner Health, Department of Clinical Research, New Orleans, Louisiana; ‡ Georgetown University, School of Medicine, Washington D.C.; § Oregon Health & Sciences University, Department of Internal Medicine, Portland, Oregon; ∥ Ochsner Health, Office of Epidemiology and Biostatistics, New Orleans, Louisiana

## Abstract

**Objectives:**

Vaccine hesitancy has been a barrier to achieving herd immunity during the coronavirus 2019 (COVID-19) pandemic. Having low socioeconomic status and education levels, and being a person of color, are associated with higher COVID-19 infection risk and worse outcomes. These same groups are associated with higher vaccine hesitancy. The state of Louisiana has one of the lowest vaccination rates in the country. In this study we aimed to identify demographic, perspective, and health behavior factors associated with vaccine hesitancy in emergency departments (ED) in Southeast Louisiana.

**Methods:**

A cross-sectional survey was distributed at three tertiary-care hospital EDs. Patients >18 years old and not in acute distress were recruited between April–July 2021. The 37-item questionnaire addressed socioeconomic demographics, social determinants of health, COVID-19 safety practices, thoughts and perceptions on COVID-19 and vaccines, sources of COVID-19 and vaccine information, and trust in the healthcare system.

**Results:**

Overall, 247 patients completed our survey. Of those, 29.6% reported they were vaccine hesitant. These respondents were significantly more likely, when compared to vaccine-acceptant respondents, to never have married, to have some college education, make less than <$25,000 in household earnings yearly, be unsure whether vaccines prevent disease, not have discussed the COVID-19 vaccine with their primary care doctor, and to prefer to do their own research for COVID-19 vaccine information. We observed no statistically significant differences based on gender, race/ethnicity, parental status, area of living, or their perceived risk of needing hospitalization for treatment or dying from the virus.

**Conclusion:**

Vaccine hesitancy was associated with multiple socioeconomic factors, perspectives, and beliefs. Vaccine-hesitant individuals were more uncertain about the safety of the COVID-19 vaccine, the feasibility of obtaining the vaccine, and its efficacy. Public health interventions aimed at these findings and improving public trust in healthcare systems are needed to increase vaccine acceptance.

## INTRODUCTION

As of April 2022, the number of global severe acute respiratory syndrome coronavirus-2 (SARS-CoV-2) cases surpassed 486 million, with over 6.1 million deaths. The United States has more cases than any other country, with nearly 79 million confirmed cases reported and 972,000 deaths.^1^ Despite the development of multiple vaccines for SARS-CoV-2, the coronavirus 2019 (COVID-19) pandemic continues to spread across the globe.

The COVID-19 vaccine rollout began in the United States in December 2020 with the US Food and Drug Administration (FDA) emergency-use authorization of the Pfizer and Moderna vaccines. Currently, 77.0% of individuals living in the US have received their first dose of the vaccine, and 65.6% are fully vaccinated.^2^ The number of vaccinations, however, is inconsistent across the US as there is widespread reluctance to receive a vaccine, also known as vaccine hesitancy.^3^

Vaccine hesitancy is not a new phenomenon. It dates back to the 1800s with the introduction of the smallpox vaccine and has played a factor in several vaccine rollouts thereafter, including diphtheria, tetanus, pertussis, mumps, and polio.^4^ An Associated Press poll in May 2020 found that only 50% of US residents reported an intent to receive the COVID-19 vaccine once available.^5^ While breakthrough cases have been reported,^6^ receiving a vaccine remains the most effective way individuals are protected from COVID-19.^7^ ^–^ ^9^ Identifying and mitigating factors related to vaccine hesitancy is crucial to increasing vaccination rates. Vaccine hesitancy can be attributed to multiple factors including the rapid development of novelty mRNA vaccines.^10^ ^,^ ^11^ Misinformation spread via social media platforms is also a contributing factor.^12^ ^,^ ^13^ Several studies found that persons with low socioeconomic status, low levels of education, being a person of color, and living in a rural area are associated with vaccine hesitancy as well.^14^ ^–^ ^17^ Consequently, these factors are also associated with higher risk of COVID-19 infection and poorer outcomes.^18^ ^–^ ^21^

Research in COVID-19 vaccine hesitancy remains limited. While papers early in the COVID-19 pandemic evaluated factors related to the intent of becoming vaccinated, few have investigated hesitancy since the vaccine became available. It is important to note that reported intentions may not always correspond with vaccine uptake.^22^ Additionally, prior vaccine-hesitancy studies focused on nationwide data. Vaccination hesitancy gaps exist among geographic locations, with the states having the most vaccine-hesitant residents concentrated in the Southeast, Midwest, and Alaska, and the least hesitant concentrated in the West and New England.^23^ The state of Louisiana has one of the lowest rates of vaccinated residents in the US, with 53.0% of its population fully vaccinated, compared to the national average of 65.6%.^24^ Given these geographic gaps, we sought to identify the demographic factors, perspectives, beliefs, and health behaviors related to vaccination hesitancy in patients presenting to emergency departments (ED) in Southeast Louisiana.

Emergency departments routinely treat the most vulnerable patient populations, including those with higher levels of adverse social determinants of health and minority communities.^25^ ^–^ ^27^ Notably, these populations are historically the most vaccine hesitant.^28^ ^–^ ^30^ By identifying determinants of COVID-19 vaccine hesitancy in the ED, public health campaigns can tailor communication efforts to address the concerns of the unvaccinated. To date, this is the first ED-based, in-person survey that investigates vaccine hesitancy as it relates to trust in the medical system. We also expand on current ED literature on COVID-19 vaccine hesitancy and social health behaviors.

## METHODS

This manuscript adheres to the EQUATOR guideline, Consensus-Based Checklist for Reporting of Survey Studies.^31^

### Study Design and Population

We conducted this cross-sectional study in the EDs at three tertiary-care hospitals within a multi-hospital system. The study was approved by our organization’s institutional review board. The questionnaire used for this research was developed using expert knowledge in emergency medicine, COVID-19, and public health, following extensive literature review. The questionnaire (Appendix 1) contains 37 questions within two sections:

• Section 1: Questions designed to collect self-reported socioeconomic demographics and social determinants of health.
• Section 2: Questions regarding COVID-19 safety practices, the respondents’ thoughts on and perceptions of COVID-19 specific vaccines, self-perceived risk, trust of the healthcare system, and sources of COVID-19 vaccine information.

Questionnaires were multiple choice but did include space for additional information if the provided answers were insufficient to the participant.

Surveys were administered in the ED between April–July 2021 by trained research staff following verbal consent. Participants were asked to participate and had the option to complete the survey on paper. Additional research information and relevant contacts were included in a cover page and provided to the participant. The completed questionnaires were transferred to and managed using REDCap, (Research Electronic Data Capture) hosted at Ochsner Main Campus, Ochsner Baptist, and Ochsner Kenner. Source documents were stored securely on site. REDCap is a secure, web-based software platform designed to support data capture for research studies.^32^ ^,^ ^33^ Only authorized, IRB-approved study team members extracted research data from source documents, entered it into the research database, and/or accessed secure patient information.

During the periods of data collection, research staff approached all adults who checked in to the ED and completed triage. Patients were excluded if they were 1) not in the waiting room of the ED, 2) were in any clear and apparent distress per discretion of research staff, or 3) had any impaired decision-making ability. If any participants needed accommodation secondary to illiteracy or visual deficiencies, a research staff member was available to read and record answers with the patient. During the research period, we did not have any participants who required additional accommodations. Patients were chosen to participate based on convenience sampling.

### Survey Context and Administration

Originally, the surveys were to be offered to patients by ED registration and nursing staff following triage at seven sites in SE Louisiana. When using this protocol, there were low rates of participation. Adjustments to the research protocol were made and surveys were only collected by available research staff at limited sites. We used ED sites for this research to collect a diverse sample of the SE Louisiana region. Table 1 reports patient demographics of the research sites, using 2020 data. Demographics of the study population are recorded in Table 2.

**Table 1.** Emergency department patient demographics.

	All sites^*^	ED #3	ED #2	ED #1
Variable	N (%)	n (%)	n (%)	n (%)
Population served	77,573	20,924	17,714	43,527
Gender				
Male	34,437 (44.39)	9,343 (44.65)	6,738 (38.04)	20,248 (46.52)
Female	43,122 (55.59)	11,580 (55.34)	10,971 (61.93)	23,271 (53.46)
Unknown/Other	14 (.02)	1 (.01)	5 (.03)	8 (.02)
Race/Ethnicity				
White	36,638 (47.23)	10,839 (51.80)	5,224 (29.49)	22,419 (51.51)
Black	36,485 (47.03)	8,331 (39.82)	11,841 (66.84)	18,908 (43.44)
Non-Black minority	2,744 (3.54)	1,329 (6.35)	313 (1.77)	1,012 (2.32)
Unknown	1,706 (2.20)	425 (2.03)	336 (1.90)	1,188 (2.73)

^*^ Not an accumulation of all three sites, patients may be counted in the demographic statistics at more than one site.

*ED*, emergency department.

**Table 2.** Participant sociodemographics.

Variable	Total sample N = 247 N (%)	Vaccine accepting n = 173 n (%)	Vaccine hesitant n = 73 n (%)	*P*-value
Age (mean years of age)	46.9	52.1	33.9	**<0.001**
Gender				0.4
Male	82 (36.0)	59 (36.2)	23 (35.4)	
Female	144 (63.2)	103 (63.2)	41 (63.1)	
Race/Ethnicity				0.2
White	77 (34.2)	60 (37)	17 (27.0)	
Black	126 (56.0)	85 (52.5)	41 (65.1)	
Non-Black minority	22 (9.8)	17 (10.5)	5 (7.9)	
Marital status				**<0.001**
Never married	79 (35.8)	44 (28.0)	34 (54.0)	
Living with partner	24 (10.9)	14 (8.9)	10 (15.9)	
Married	69 (31.2)	59 (37.6)	10 (15.9)	
Divorced or separated	39 (17.7)	32 (20.4)	7 (11.1)	
Widowed	10 (4.5)	8 (5.1)	2 (3.2)	
Children				0.3
Have children	154 (71.0)	114 (73.1)	40 (65.6)	
No children	63 (29.0)	42 (26.9)	21 (34.4)	
Education				**0.04**
Some education but non-high school graduate	27 (12.1)	16 (10.1)	10 (15.6)	
High school graduate	55 (24.6)	36 (22.6)	19 (29.7)	
Some college/university	65 (29.0)	43 (27.0)	22 (34.4)	
College/university graduate or above	77 (34.4)	64 (40.3)	13 (20.3)	
Employment status				**<0.001**
Working	120 (53.3)	86 (53.1)	34 (54.0)	
Retired	46 (20.4)	43 (26.5)	3 (4.8)	
Laid off	20 (8.9)	9 (5.6)	11 (17.5)	
Other	39 (17.3)	24 (14.8)	15 (23.8)	
Average household income				**0.003**
<$25,000	88 (43.1)	58 (40.9)	30 (48.4)	
$25,000−$74,999	73 (35.8)	45 (31.7)	28 (45.2)	
≥$75,000	43 (21.1)	39 (27.5)	4 (6.5)	
Area of living				0.2
Small town/rural	35 (15.9)	24 (15.4)	11 (17.5)	
Suburban	42 (19.1)	35 (22.4)	7 (11.1)	
City	143(65.0)	97 (62.2)	45 (71.4)	
Political orientation				**0.002**
Republican	31 (14.6)	23 (14.9)	8 (13.6)	
Democrat	95 (44.6)	82 (53.3)	13 (22.0)	
Libertarian	3 (1.4)	2 (1.3)	1 (1.7)	
Green	0 (0)			
Independent	20 (9.4)	12 (7.8)	8 (13.6)	
No political orientation	27 (12.7)	15 (9.7)	12 (20.3)	
Prefer not to answer	33 (15.5)	18 (11.7)	15 (25.4)	
Other	4 (1.7)	2 (1.3)	2 (3.4)	

The optimal sample size for this research based on a population of approximately 80,000 patients served at the three ED sites was 400 participants, calculated using a 5% margin of error and 95% confidence interval. However, a high non-participation rate was expected per literature review on similar research.^34^ Additionally, due to the third and fourth wave of COVID-19 and the emergence of the delta variant, we stopped survey collection with a sample size of 294 to keep research conditions relatively constant.

### Data Analysis

We used means, standard deviations, frequencies, and percentages to describe the cohort’s sociodemographic characteristics, opinions and health behaviors related to COVID-19 and vaccines. Respondents were categorized as *vaccine hesitant* if they answered “No” or “Unsure” to the question: “Do you plan to receive a COVID-19 vaccine?” and as *vaccine accepting* if they answered “I have already received the vaccine” or “Yes” to the same question. We examined comparisons of respondents’ sociodemographic characteristics, opinions and health behaviors related to the COVID-19 virus and vaccines between the vaccine-hesitant and vaccine-accepting groups with *t*-tests, chi-square, or Fisher exact tests. We used SAS version 9.4 (SAS Institute Inc, Cary, NC) to perform all analyses.

## RESULTS

Overall, 247 patients participated in our survey, with most responses coming from ED #1 (115) and ED #2 (105). Tables 2–4 describe the results of the demographic, perspective/opinions, and health behavior portions of the questionnaire. Of those who participated, 246 answered our primary question, “Do you plan to receive the COVID-19 vaccine?”; 70.3% indicated that they planned to receive or had already received the COVID-19 vaccine and 29.55% reported they had no plans to receive the vaccine or were unsure whether they were going to receive the vaccine. Most participants in this study were female (63.2%), Black (56.0%), never married (35.7%), were parents (71.0%), employed (53.3%), had a household income of <$25,000, and lived in the city (65.0%) (Table 2).

**Table 3.** Perspective/opinion questions and responses.

	Total sample	Vaccine accepting	Vaccine hesitant	
Variable	N (%)	n (%)	n (%)	*P*-value
When available to you, how difficult do you think it will be to get access to the COVID-19 vaccine?				**<0.001**
Very/somewhat difficult	5 (2.1)	2 (1.2)	3 (4.2)	
Neutral	8 (3.4)	4 (2.4)	4 (5.6)	
Easy/Very easy	71 (30.0)	35 (21.2)	36 (50)	
Unsure	35 (14.8)	6 (3.6)	29 (40.3)	
I have already received the COVID-19 vaccine	118 (49.8)	118 (71.5)	0 (0)	
What do you think are your chances of being infected by the COVID-19 virus?				**0.01**
Low	186 (77.2)	138 (82.1)	47 (65.3)	
Medium	43 (17.8)	22 (13.1)	21 (29.2)	
High	12 (5.0)	8 (4.8)	4 (5.6)	
What do you think are your chances of needing to be hospitalized for treatment for COVID-19?				0.1
Low	212 (88.0)	152 (90.5)	59 (81.9)	
Medium	20 (8.30)	12 (7.1)	8 (11.1)	
High	9 (3.7)	4 (2.4)	5 (6.9)	
What do you think are your chances of dying from COVID-19 virus				0.2
Low	217 (92.0)	157 (94.0)	60 (87.0)	
Medium	10 (4.2)	6 (3.6)	4 (5.8)	
High	9 (3.8)	4 (2.4)	5 (7.3)	
What is your best guess as to whether you will get the coronavirus within the next 6 months?				1.0
I don’t think I will get the coronavirus.	196 (83.1)	138 (82.6)	58 (84.1)	
I think I will get a mild case of the coronavirus.	17 (7.2)	12 (7.2)	5 (7.3)	
I think I will get seriously ill from the coronavirus.	17 (7.2)	13 (7.8)	4 (5.8)	
I already had the coronavirus, and I don’t think I will get it again.	6 (2.5)	4 (2.4)	2 (2.9)	
Which best describes your overall state of health?				**0.02**
Great health	41 (17.3)	21 (12.6)	20 (28.6)	
Good health	88 (37.1)	63 (37.7)	25 (35.7)	
Average health	86 (36.3)	65 (38.9)	21 (30)	
Poor health	22 (9.3)	18 (10.8)	4 (5.7)	
Do you believe vaccines, in general, help prevent disease?				**<0.001**
Yes	168 (70.6)	147 (87.0)	21 (30.4)	
No	25 (10.5)	4 (2.4)	21 (30.4)	
Unsure	45 (18.9)	18 (10.7)	27 (39.1)	
Do you believe vaccines, in general, are harmful?				**<0.001**
Yes	28 (11.6)	14 (8.2)	13 (18.8)	
No	153 (63.5)	125 (73.1)	28 (40.6)	
Unsure	60 (24.9)	32 (18.7)	28 (40.6)	
Do you believe the COVID-19 vaccine can prevent COVID-19 disease?				**<0.001**
Yes	121 (51.1)	115 (68.5)	5 (7.4)	
No	34 (14.4)	6 (3.6)	28 (41.2)	
Unsure	82 (34.6)	47 (28.0)	35 (51.5)	
Do you think you have enough information to make a decision on the COVID-19 vaccine?				**<0.001**
Yes	187 (79.9)	157 (92.9)	30 (46.2)	
No	47 (20.1)	12 (7.1)	35 (53.9)	
Preferred method of receiving COVID-19 vaccine information				
Discussion with healthcare practitioner	84 (34.0)	59 (34.1)	24 (32.9)	0.9
Pamphlets, flyers, articles	16 (6.5)	12 (6.9)	4 (5.5)	0.7
Videos	14 (5.7)	9 (5.2)	5 (6.9)	0.6
Own research	27 (10.9)	9 (5.2)	18 (24.7)	**<0.001**
Waiting to see how others do after being vaccinated	25 (10.1)	8 (4.6)	17 (23.3)	**<0.001**
Discussion with people who are vaccinated	31 (12.6)	20 (11.6)	11 (15.1)	0.5
Other	14 (5.7)	13 (7.5)	1 (1.4)	0.1
Unsure	13 (5.3)	4 (2.3)	9 (12.3)	**0.001**
Do you trust that healthcare practitioners have your best interest in mind when recommending the COVID-19 vaccine?				**<0.001**
Yes	185 (78.4)	154 (90.6)	31 (47.0)	
No	13 (5.5)	4 (2.4)	9 (13.6)	
Unsure	38 (16.1)	12 (7.1)	26 (39.4)	

*COVID-19*, coronavirus 2019.

**Table 4.** Health behavior questions and responses.

	Total sample	Vaccine accepting	Vaccine hesitant	
Variable	N (%)	n (%)	n (%)	*P*-value
Have you had a positive test for COVID-19?				0.2
Yes	43 (17.7)	35 (20.5)	8 (11.1)	
No	183 (75.3)	124 (72.5)	59 (81.9)	
I have never been tested for COVID-19	17 (7.0)	12 (7.0)	5 (6.9)	
Do you generally wear masks in public and around other people?				**0.03**
Yes	224 (91.1)	162 (93.6)	62 (84.9)	
No	22 (8.9)	11 (6.4)	11 (15.1)	
How many people do you interact with, mask-less and without social distancing, in a typical week?				0.1
0	35 (14.3)	26 (15.3)	8 (11.0)	
Between 1 to 5	123 (50.4)	85 (50)	38 (52.1)	
Between 6 to 10	48 (19.7)	37 (21.8)	11 (15.1)	
Between 11 to 20	11 (4.5)	5 (2.9)	6 (8.2)	
Between 21 to 30	6 (2.5)	2 (1.2)	4 (5.5)	
30 or more	21 (8.6)	15 (8.8)	6 (8.2)	
In the past week, how often did you practice social distancing, that is, you maintained a distance of at least 6 feet between you and other people?				0.7
Never	16 (6.5)	9 (5.3)	6 (8.2)	
Some of the time	50 (20.4)	33 (19.3)	17 (23.3)	
Most of the time	87 (35.5)	62 (36.3)	25 (34.3)	
All the time	92 (37.6)	67 (39.2)	25 (34.3)	
Do you have medical insurance?				0.3
Yes	211 (89.8)	153 (91.1)	58 (86.6)	
No	21 (8.9)	14 (8.3)	7 (10.5)	
Unsure	3 (1.3)	1 (0.6)	2 (3.0)	
Did you get the flu vaccine last year?				**<0.001**
Yes	134 (55.8)	109 (64.1)	25 (36.2)	
No	105 (43.8)	61 (35.9)	43 (62.3)	
Unsure	1 (0.4)	0 (0)	1 (1.5)	
Do you have a primary care doctor?				0.4
Yes	201 (84.5)	145 (86.3)	55 (79.7)	
No	31 (13.0)	20 (11.9)	11 (15.9)	
Unsure	6 (2.5)	3 (1.8)	3 (4.4)	
If yes, have you discussed the COVID-19 vaccine with your primary care doctor?				**<0.001**
Yes	125 (63.8)	105 (73.4)	20 (30.7)	
No	71 (36.2)	38 (26.6)	33 (62.3)	

*COVID-19*, coronavirus 2019.

Among sociodemographic characteristics, we found significant associations between vaccine hesitancy and age, marital status, education level, work status, and household income (*P* < 0.05). On average, vaccine-hesitant individuals were younger than those in the vaccine-acceptant cohort (33.88 vs 52.10, *P* < 0.001). Respondents who were vaccine hesitant were more likely to never have been married (53.97 vs 28.03, *P* < 0.001), to have some college/university education without graduating (34.38 vs 27.04, *P* < 0.042), were less likely to be retired (4.76 vs 26.54, *P* < 0.001), and made less than $25,000 in household earnings (48.39 vs 40.85, *P* < 0.003), compared to respondents who were vaccine acceptant. Vaccine-acceptant individuals were more likely to be Democrat (53.25 vs 22.03, *P* < 0.002). This study did not find any statistically significant differences between vaccine-acceptant and vaccine-hesitant groups based on gender, race/ethnicity, parental status, or area of living (Table 2).

Survey questions concerning perceived difficulty accessing the COVID-19 vaccine, chances of being infected with COVID-19, and overall state of health were significantly associated with vaccine hesitancy. Respondents who were vaccine hesitant were more unsure about their ease of obtaining the vaccine (40.28 vs 3.64 *P* < 0.001) and perceived a higher chance of being infected with the COVID-19 virus (29.17 vs 13.10, *P* < 0.01), compared to those who were vaccine hesitant. In general, more vaccine-hesitant individuals thought of themselves as being in great health (28.57 vs 12.57, *P* < 0.02), compared to respondents who were vaccine acceptant. There were no significant associations between vaccine hesitancy and perceived risk of contracting the virus in the following six months, needing hospitalization for treatment, or dying from the virus (Table 3).

We found significant associations between vaccine hesitancy and perceived vaccine effectiveness. Vaccine-hesitant respondents did not believe that vaccines in general help prevent disease (30.43 vs 2.37, *P* < 0.001) and believed in general that vaccines were harmful (40.58 vs 18.71, *P* < 0.001), compared to non-vaccine-hesitant respondents. Vaccine-hesitant individuals were more likely to be unsure whether the COVID-19 vaccine prevented COVID-19 disease (51.47 vs 27.98, *P* < 0.001), compared to those who were not vaccine hesitant.

Vaccine-hesitant respondents believed they did not have enough information to decide on the COVID-19 vaccine (53.85% vs 7.10%, *P* < 0.001) and preferred to receive COVID-19 vaccine information by doing their own research (24.66% vs 5.20%, *P* < 0.001) or waiting to see how others reacted after being vaccinated (23.29% vs 4.62%, *P* < 0.001) (Table 3). Vaccine-hesitant respondents were more unsure whether healthcare clinicians had their best interests in mind when recommending the COVID-19 vaccine (39.39% vs 7.06%, *P* < 0.001), compared to vaccine-acceptant individuals. There was no significant difference in other forms of receiving COVID-19 vaccine information (Table 3).

Vaccine-hesitant individuals were less likely to wear masks in public (84.93% vs 93.64%, *P* < 0.029) and to have gotten the flu vaccine the previous year (36.23% vs 64.12%, *P* < 0.001), compared to vaccine-acceptant individuals (Table 4). Furthermore, vaccine-hesitant respondents were less likely to have discussed the COVID-19 vaccine with their primary care doctor (30.74% vs 73.43%, *P* < 0.001), compared to vaccine-acceptant individuals (Table 4).

We did not find associations between vaccine hesitancy and previous positive COVID-19 test, social distancing, or having a primary care doctor. There was also no statistically significant difference between vaccine-acceptant and vaccine-hesitant participants regarding medical insurance status and the number of people respondents interacted with mask-less (Table 4).

## DISCUSSION

Currently, approximately 65% of the US population is fully vaccinated against COVID-19.^2^ Although the national vaccination rate has improved, the local vaccination rate at the state level lags in certain areas. Louisiana has one of the lowest vaccination rates (53%) in the country and one of the highest mortality rates secondary to COVID-19.^35^ The vaccine gap threatens to unnecessarily prolong the COVID-19 pandemic. This study demonstrates an association between vaccine hesitancy and multiple demographic factors, health attitudes, opinions, and behaviors.

The majority of our respondents self-identified as Black and female and had an average age of 46. (Table 2). Prior research shows a connection between Black race, female gender identity, and vaccine hesitancy.^36^ ^–^ ^38^ The lack of race and gender association seen in this investigation could be due to the small census numbers across multiple ethnicities. Larger studies using electronically distributed surveys show differences based on ethnicity and race.^39^ We halted our study prematurely due to the higher risk of exposure during the Sars-CoV-2 delta-variant surge. Further subgroup analysis was considered; however, smaller sample sizes make results less generalizable. Moreover, the intersectionality of gender and race was not investigated in this study. Previous research shows higher vaccine hesitancy in respondents who identify as both Black and female compared to others.^39^ Larger surveys in the future could evaluate subgroup associations with vaccine hesitancy in men and women of different ethnicities and races.

The observations in this research are consistent with prior studies finding that vaccine-hesitant individuals were younger than the vaccine acceptant (Table 2). The difference in overall mortality and morbidity of COVID-19 seen across ages may explain this discrepancy. Older patients have worse outcomes, higher risk of hospitalization, and higher risk of death compared to younger patients.^40^ ^,^ ^41^ Potentially, younger individuals believe they are at lower risk for worse outcomes and, therefore, do not see a need for vaccination. Even though younger individuals have a lower risk of severe disease, the risk is not zero. Additionally, younger patients can still serve as asymptomatic carriers and infect susceptible friends and family. A message tailored to younger populations focusing on the hazards of transmitting the virus to higher risk friends and family should be a public health goal.

The research on vaccine hesitancy and marital status is unclear. This study documents an association between vaccine hesitancy and respondents who were never married (Table 2). Prior studies show an association between being in a relationship and vaccine hesitancy.^42^ Conflicting research has shown that married couples were more likely to accept the vaccine.^36^ ^,^ ^43^ Married people engage in healthier daily behaviors and live longer lives compared to unmarried.^44^ ^,^ ^45^ It is possible that having a significant other provides a healthier support network and pressure to retain healthier behaviors. This could also be explained by nepotism; however, our study did not demonstrate an association with vaccine acceptance and having children (Table 2). This is puzzling as one would think having children to care for would convince respondents to get the vaccine either for one’s own well-being or to reduce the risk of transmitting the virus to family members. Conceivably the low morbidity and mortality in the pediatric population had study participants less concerned about transmitting the virus to younger children.^46^ ^,^ ^47^

Similar to prior studies, lower household income and education levels were associated with vaccine hesitancy. Multiple socioeconomic factors may influence overall health literacy.^36^ ^,^ ^42^ ^,^ ^48^ Lower levels of education may result in a decreased chance of learning and developing skills necessary to critically appraise health information.^20^ ^,^ ^36^ ^–^ ^38^ ^,^ ^42^ ^,^ ^46^ ^,^ ^47^ Both lower education levels and lower income can lead to fewer opportunities to understand health information and less access to health care.^49^ ^,^ ^50^ Additionally, lower education levels may cause individuals to be more easily swayed by misinformation.^51^ The ED often offers the most timely access to the healthcare system for vulnerable populations in lower socioeconomic classes.^52^ ^,^ ^53^ The ED is a prime location to intervene and offer educational materials and teachings about the COVID-19 vaccine.

Political affiliation is strongly correlated with vaccine acceptance.^54^ People who identify as Democrat are more likely to be vaccine acceptant while Republicans are more likely to be vaccine hesitant. This study found Democrats to be vaccine acceptant but lacked the hesitant association with Republicans. A portion of respondents preferred not to answer, which could have affected outcomes. Additionally, respondents may have been apprehensive about sharing their political affiliation given the current, divisive political climate or they feared it could have affected the quality of their care.

Individual attitudes, beliefs, and perceptions are the most influential predictors of vaccine acceptance.^55^ Our study expands on the 2021 work of Fernandez-Penny et al, which delved into vaccine hesitancy as it relates to attitudes/perceptions of the COVID-19 virus and disease and trust in the medical system. Vaccine-hesitant individuals in this survey felt they were in better health compared to vaccine-acceptant individuals, which falls in line with previous studies.^56^ Presumably if respondents believed they were in good or great health, they did not consider themselves to be at risk of being hospitalized or dying from COVID-19 disease and, therefore, did not wish to have the vaccine.

Equitable vaccine access is one of the cornerstones of proper vaccine distribution. Hospitals throughout the nation have formed health equity committees to ensure equitable allocation. Despite the number of vaccine distribution centers in SE Louisiana, respondents to this survey were unsure of their ability to access the vaccine. One potential reason is that vaccine distribution centers were not set up in the areas of greatest need. Access can be stifled by geographic barriers. Low socioeconomic areas have been overlooked while organizing vaccine distribution centers around the country.^57^ A second reason behind perceived poor access could be a lack of advertisement of existing distribution centers in these areas. To meet the needs of the community, planned access and equitable distribution of vaccine centers should be organized with community engagement in mind.

One of the most prevalent reasons for vaccine hesitancy is the perceived overall safety of the vaccine.^34^ ^,^ ^38^ ^,^ ^42^ Respondents were unsure whether vaccines in general were harmful. Many believe that the COVID-19 vaccine was developed too quickly, bypassing safety protocols for economic incentives.^34^ Although the COVID-19 vaccine is novel, the technological and scientific basis of the vaccine has been well studied.^58^ Strategies for public education regarding vaccine safety should consider communication surrounding unprecedented global partnership and rigorous testing before and during vaccine rollout.

This investigation was performed during the advent of the SARS-CoV-2 delta variant. Despite the increased transmissibility of the delta variant, most respondents believed they had a low chance of contracting the SARS-CoV-2 virus.^59^ Vaccine-hesitant individuals believed they had a higher chance of contracting the virus compared to the vaccine acceptant. One explanation behind this discrepancy is that the vaccine-acceptant group may contain individuals who have already gotten the vaccine. These same individuals believe in the protective effects of the vaccine and perceive a lower chance of contracting the virus. Also, despite a perceived higher risk of contracting the virus, most vaccine-hesitant respondents did not believe that vaccines prevented disease. These results are in line with prior vaccine-hesitancy literature.^37^ ^,^ ^38^ ^,^ ^59^ ^,^ ^60^ Public health interventions may need to focus on the clearly established benefit vs very low risk of vaccination, while also highlighting the effectiveness of COVID-19 vaccines in preventing hospitalization and death.^61^

The survey findings show respondents who were vaccine hesitant would like to do more of their own research or wait until others have had the vaccine before getting it themselves (Table 3). COVID-19 vaccine information has been distributed in multiple formats. Public health advocates should focus on continuously disseminating information on the vaccine in different formats to encourage vaccine uptake. Social media, for example, is an important avenue to encourage positive health behaviors.^62^ Hospitals could partner with organizations that cater to at-risk and socially vulnerable populations to form creative educational resources.

This is the first ED-based, in-person survey study to measure vaccine hesitancy as a dependent factor of medical mistrust. Respondents who were vaccine hesitant did not believe that health practitioners had their best interest in mind when recommending the vaccine (Table 3). Multiple media outlets falsely reported that clinicians were taking monetary incentives for inappropriately diagnosing COVID-19 infections and for distributing the vaccine.^63^ ^,^ ^64^ Medical mistrust, in certain populations, is based on years of mistreatment by the medical community. Healthcare professionals should be given the tools to suspend judgment regarding the vaccine hesitant and understand the historical, political, and social context that has disproportionally disparaged vulnerable populations. Public health officials may need to rethink ideas of encouraging vaccine acceptance by investing in ways to build trust within the medical community.^65^ Emergency physicians can promote changes in health behaviors and should use their limited time to engage in a patient-centered discussion on the utility of vaccines.^66^

This study adds to the current research on vaccine hesitancy in the ED setting. The ED is a unique context as it serves vulnerable populations. The COVID-19 pandemic has preferentially affected racial minorities and people in a lower socioeconomic class. Prior research shows these same populations are less likely to accept the vaccine. Our investigation can help elucidate target populations to deliver health messages. A televised public health intervention using health practitioners could grow more vaccine acceptance over time. In the ED, vaccination discussions during ED visits give health access to lower socioeconomic classes and provides an opportunity to speak with a clinician. Continuing an ED-based vaccination effort could increase the proportion of vaccinated vulnerable peoples.

Future directions could expand on our research by using longitudinal survey and logistical regression models. Previous studies support the transtheoretical model of change: behavioral change does not occur at one point but at various stages in a cycle.^67^ Vaccine hesitancy is a labile trait and can change overtime.^68^ Longitudinal studies can discover changing opinions over time with each variant surge and the need for further booster shots. Additionally, discovering the strength of association with vaccine hesitancy and changing opinions can help tailor public health interventions. Future survey studies can also focus on a more diverse group of respondents. Occupations that place individuals at higher risk of COVID-19 disease are often performed by economically and socially disadvantaged populations.^20^ Prior investigations highlight that these same individuals are more vaccine hesitant. Workers in the healthcare sector can organize interventions that speak to these communities to obtain novel perspectives.

## LIMITATIONS

The present study is not without limitations. The survey was conducted in person with patients in the ED. Social desirability bias may have influenced our study as it took place in a healthcare facility, around health practitioners, during a time when the vaccine became more widely available. Respondents may have wanted to be falsely agreeable to vaccine acceptance while they were in a healthcare setting. This research focused on vaccination hesitancy solely in the adult population. The pediatric vaccination rate is lower than the adult rate in some age groups in Louisiana.^69^ The reasons surrounding pediatric vaccination hesitancy may not coincide with that of the adult population. This study was also limited to the answer choices we provided in the survey, which did not give space for novel perspectives, attitudes, and opinions from our respondents. A qualitative or a mixed-methods approach could reveal new factors associated with vaccine hesitancy that have not yet been published.

Geographic context is associated with vaccine hesitancy. Our study took place at three different sites. Few respondents were taken from ED #3, a location at a considerable distance from a major city that was noted to have lower vaccination rates.^69^ This may have influenced the results of the survey as each hospital was not represented proportionately.

## CONCLUSION

This study demonstrates that, despite the finding that vaccine-hesitant patients perceive a higher risk of contracting COVID-19, they feel more uncertain about the safety of the COVID-19 vaccine, the feasibility of obtaining the vaccine, and its efficacy. Many vaccine-hesitant patients felt as though they did not have enough information to make the decision to accept or decline the COVID-19 vaccine, while at the same time many preferred to do their own research and were unsure how much trust to place in their physicians. Further studies should focus on what platforms could be used and trusted by this patient population to provide scientifically sound education to the vaccine-hesitant population.
